# Incidence of pelvic high-grade serous carcinoma after isolated STIC diagnosis: A systematic review of the literature

**DOI:** 10.3389/fonc.2022.951292

**Published:** 2022-08-31

**Authors:** Valerie Catherine Linz, Amelie Löwe, Josche van der Ven, Annette Hasenburg, Marco Johannes Battista

**Affiliations:** Department of Gynaecology and Obstetrics, University Medical Centre, Johannes Gutenberg University Mainz, Mainz, Germany

**Keywords:** serous tubal intraepithelial carcinoma (STIC), high-grade serous carcinoma (HGSC), precursor, peritoneal carcinomatosis, incidence, treatment, outcome

## Abstract

**Objective:**

Serous tubal intraepithelial carcinoma (STIC) is a precursor lesion of pelvic high-grade serous carcinoma (HGSC). Information on treatment and outcome of isolated STIC is rare. Therefore, we reviewed systematically the published literature to determine the incidence of subsequent HGSC in the high- and low-risk population and to summarize the current diagnostic and therapeutic options.

**Methods:**

A systematic review of the literature was conducted in MEDLINE-Ovid, Cochrane Library and Web of Science of articles published from February 2006 to July 2021. Patients with an isolated STIC diagnosis and clinical follow-up were included. Study exclusion criteria for review were the presence of synchronous gynaecological cancer and/or concurrent non-gynaecological malignancies.

**Results:**

3031 abstracts were screened. 112 isolated STIC patients out of 21 publications were included in our analysis with a pooled median follow-up of 36 (interquartile range (IQR): 25.3-84) months. 71.4% of the patients had peritoneal washings (negative: 62.5%, positive: 8%, atypic cells: 0.9%). Surgical staging was performed in 28.6% of all STICs and did not show any malignancies. 14 out of 112 (12.5%) patients received adjuvant chemotherapy with Carboplatin and Paclitaxel. Eight (7.1%) patients developed a recurrence 42.5 (IQR: 33-72) months after isolated STIC diagnosis. Cumulative incidence of HGSC after five (ten) years was 10.5% (21.6%). Recurrence occurred only in *BRCA1* carriers (seven out of eight patients, one patient with unknown *BRCA* status).

**Conclusion:**

The rate of HGSC after an isolated STIC diagnosis was 7.1% with a cumulative incidence of 10.5% (21.6%) after five (ten) years. HGSC was only observed in *BRCA1* carriers. The role of adjuvant therapy and routine surveillance remains unclear, however, intense surveillance up to ten years is necessary.

**Systematic Review Registration:**

https://www.crd.york.ac.uk/prospero/, identifier CRD42021278340.

## Introduction

Serous tubal intraepithelial carcinoma (STIC) in the fimbriated end of the fallopian tube is regarded as the precursor lesion of pelvic (i.e. ovarian or peritoneal) high-grade serous cancer (HGSC) (1–3). Women with proven *BRCA* germline mutations have an increased risk of 10-60% for developing ovarian cancer. For these women, a risk-reducing salpingo-oophorectomy (RRSO) is therefore recommended and presents the most effective method of prevention so far (4, 5). Occult carcinoma and/or STIC is detected in approximately 10-15% of these cases (1), isolated STIC is detected in approximately 2% (6). Metachronous peritoneal carcinomatosis after RRSO in high-risk patients occurs in approximately 4.5% (7) and predominantly in *BRCA1* mutation carriers, usually within 5 years (8). Moreover, STIC diagnosis accompanies more than half of the cases with sporadic ovarian, tubal or primary peritoneal cancer (1). The incidence of STIC in patients with a normal risk of ovarian cancer is uncertain; however, a Canadian study reported STIC in eight out of 9392 women (<0.01%) with benign diagnoses (9). Accordingly, a recently published population-based, retrospective cohort study of all individuals in British Columbia, Canada, who underwent opportunistic salpingectomy or a control surgery (hysterectomy alone or tubal ligation), showed that the opportunistic salpingectomy group had significantly fewer serous and epithelial ovarian cancers than the control group (10). In the future, opportunistic salpingectomies will likely increase in routine surgery as a strategy for epithelial ovarian cancer prevention.

The SEE-FIM (Sectioning and Extensively Examining the FIMbria) protocol helps pathologists to detect these STIC lesions and is nowadays established for RRSOs after its first publication in February 2006 (11). Women with a proven isolated STIC lesion are at substantial risk to develop advanced HGSC and the metastatic pattern of a STIC remains unclear (6, 12, 13). Furthermore, consistent information on diagnostic necessities and therapeutical consequences for patients with STIC is lacking so far since most of the literature is focusing on pathological features (7).

The aim of this review was to determine the incidence of HGSC following a proven, isolated STIC diagnosis to discuss the management and follow-up of these women. Additional outcomes comprised the description of therapeutic and diagnostic options for STICs in the clinical routine.

## Methods

### Literature search and eligibility criteria

Our systematic review is based on the Preferred Reporting Items for Systematic Reviews and Meta-Analyses (PRISMA) statement (14). It is registered at PROSPERO (CRD42021278340).

Three electronic bibliographical databases including MEDLINE (*via* Ovid), the Cochrane Central Register of Controlled Trials (CENTRAL) and Web of Science were searched systematically from February 2006 to July 2021 (15). In February 2006, the SEE-FIM protocol was initially introduced to detect STICs in routine diagnostics regularly (11).

The search strategies for each database were conducted by a librarian from the Johannes Gutenberg- University Mainz according to the PICOS criteria (16). All search strategies included index terms as well as free text related to STIC. The search strategies are provided in the supplementary material (appendix A). The search was performed on 28th July 2021. Furthermore, studies included in related systematic reviews and meta-analyses were screened for eligibility. A de-duplication of database search results in EndNote was performed according to Bramer (17). Grey literature, such as conference abstracts, were not included.

Study inclusion criteria for review were the pathological diagnosis of isolated STIC and clinical follow-up. Patients with a STIC and a positive cytology were also included to maintain consistency with previous publications on this subject (7). Serous intraepithelial neoplasia is also known as STIC and was included (18). Study exclusion criteria for review were missing clinical data (follow-up) and publications restricted to pathological information only. In addition, the presence of synchronous gynaecological cancer and/or concurrent non-gynaecological malignancies were exclusion criteria. Patients with a STIC diagnosis at RRSO and with an upstaging to a HGSC at the following surgical staging were not included, since the HGSC might have been overlooked at the initial surgery. Meta-analyses, systematic reviews, literature review and case reports were not included. Results should be interpreted accordingly. Only the latest published data were reported in case of articles that were an update of previously published patients.

### Data extraction

Title and abstract screening, as well as full-text screening, were conducted by two review authors (V.C.L and A.L.) independently. A third independent reviewer (M.J.B) was contacted in case of disagreements between the first two reviewers. Data extraction was performed by V.C.L. and re-checked independently by A.L. using a predefined EXCEL spread sheet. The following information was collected: age, personal history of breast cancer, genetic predispositions, surgical indications, preoperative serum CA-125 levels, preoperative pelvic ultrasound, surgical procedure, peritoneal washings, adjuvant treatment (e.g. completion surgery, chemotherapy), and follow-up.

### Risk of bias assessment

For each cohort study adequateness was assessed by the following criteria based on a systematic review of Van der Hoeven in 2018 (19): STICs should be diagnosed according to predefined pathological criteria and by an expert pathologist. The reporting bias included the description of the original cohort size, the genetic predisposition, median or mean age at surgery, information about clinical staging and adjuvant treatment for the patients with STIC. The indication was considered adequate if the surgery and the treatment of STIC took place according to a predefined protocol. The reported follow-up was seen as adequate if the follow-up was given in months or years describing the presence or absence of recurrence.

### Data synthesis and analysis

A pooled incidence of subsequent HGSC with a corresponding confidence interval (CI) after an isolated STIC diagnosis was calculated for all patients with an isolated STIC and follow-up. The median is shown with interquartile ranges (IQR) if possible. The Kaplan-Meier estimation was used to calculate the cumulative incidence of HGSC.

Staging procedures, adjuvant treatment and their outcome were described. Due to the limited number of recurrences, a risk stratification as well as a statistical analysis of the associations between staging, chemotherapy and recurrence was not performed (19).

A statistical analysis was carried out using SPSS version 27 (SPSS, Chicago, IL, USA).

## Results

**Figure 1:**
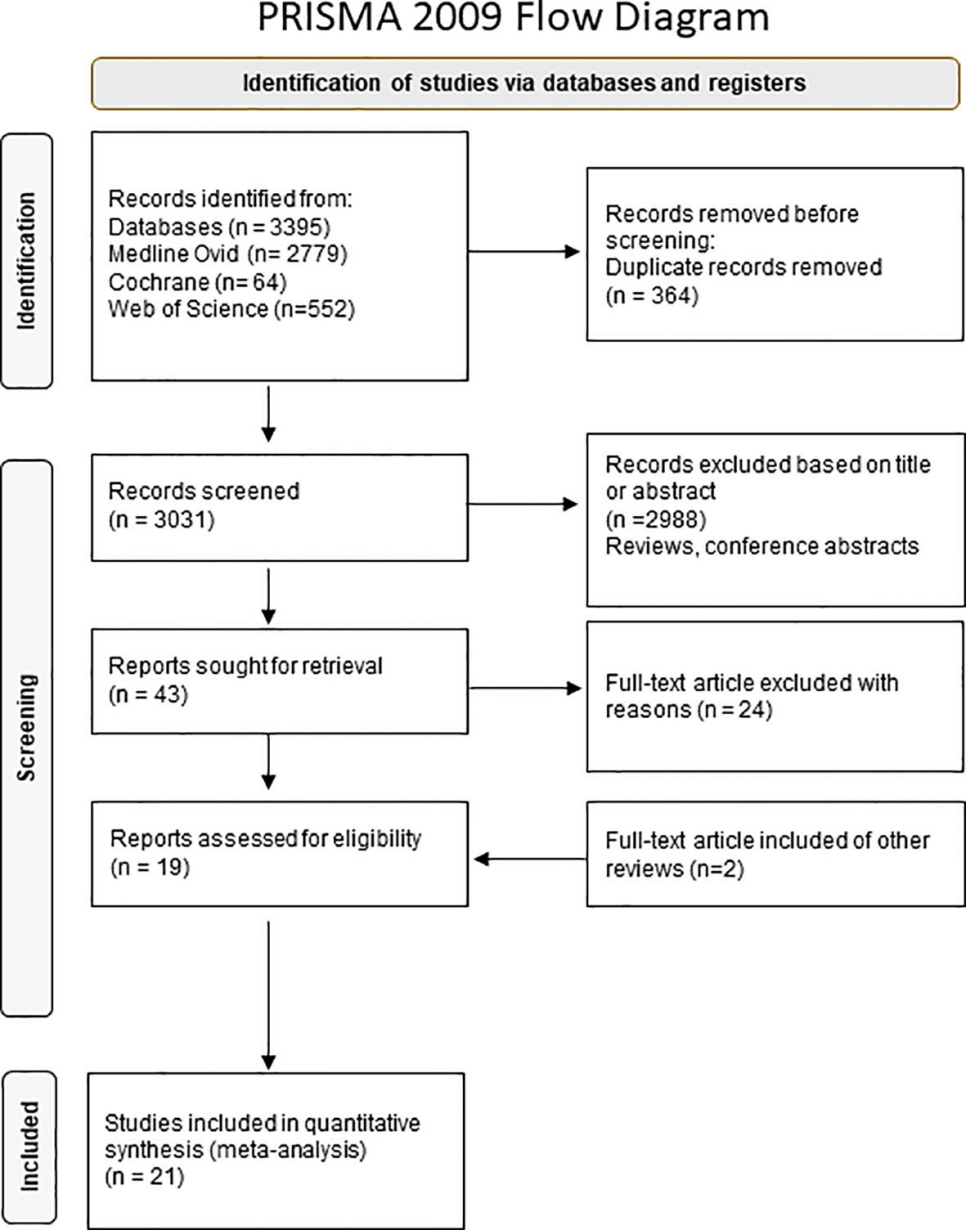
Flowchart of literature selection.

In total, 3031 records were screened and 21 articles met our inclusion criteria as shown in the PRISMA flow chart in **Figure 1**.

We were able to include 112 patients out of these 21 articles (**Table 1** and **Table 2** for detailed information; **Table 3** for overview). Median age was 52.3 (46.3-60) years. 71 (63.4%) patients were *BRCA1* carriers, 18 (16.1%) patients were *BRCA2* carriers. Eight (7.1%) patients were either *BRCA1* or *2* positive. Four patients (3.6%) had a high risk and four patients a low risk of ovarian cancer. The *BRCA* status was unknown for five (4.5%) patients. Two (1.8%) were *BRCA* negative. One patient had a *PALB2* mutation (31). RRSO was performed in 100 patients due to *BRCA* mutations or high-risk personal or family history. An opportunistic salping(o-oophor)ectomy was performed in the remaining twelve patients with an isolated STIC during surgery for benign reasons (ovarian cyst, cholecystectomy) (12, 34–36). In some cases, additional procedures were performed, mostly hysterectomies. All individual procedures are listed for each study in **Table 1**. Peritoneal washing during RRSO/surgery was reported in 80 (71.4%) cases of which nine (8%) were positive and one (0.9%) showed atypical cells. Six out of these nine patients had immediate reoperation for surgical staging. One patient declined the offer and opted for observation with CA-125 biannually and clinical review yearly for 3.5 years, and afterwards was discharged to the local medical officer (26). All of the surgical stagings showed no pathological findings and no subsequent HGSC was described in the follow-up.

**Table 1:** Detailed characteristics of all included patients with isolated STIC (white: high-risk cohort; grey: low-risk cohort).

Reference	Number of cases (STIC/total RRSO or performed surgeries)	Median age (range) or mean age in years	Previous cancer	*BRCA* status/mutation status	CA 125	Pelvic USG	Cytology outcome at RRSO/surgery	Additional procedure at RRSO/surgery and outcome
High-risk cohort								
Blok 2019 (13)	4/527	54 (47.8-67)	Breast (1)	BRCA1 (3) BRCA2 (1)	Normal (4)	Normal (4)	Negative (2) ND (2)	
Carcangiu 2006 (20)	3/50	52.7 (+/- 7.2)	Breast (3)	BRCA1 (3)	Normal (3)	Normal (3)	Negative (2) NR (1)	TAH with USO (1)
Conner 2014 (21)	11/349	49 (41-53)	Breast (2)	BRCA1 (5) BRCA2 (1) BRCA1 or 2 (5)	ND (11)	ND (11)	Negative (6) NR (5)	
Gornjec 2020 (22)	3/155	62 (+/- 8.2)	NR (3)	BRCA1 (2) High-risk (1)	Normal (3)	Normal (3)	Negative (3)	
Lamb 2006 (23)	4/113	49.5 (46.3-61.8)	NR (4)	BRCA1 (3) BRCA2 (1)	NR (4)	NR (4)	Positive (1) Negative (3)	
Miller 2017 (24)	3/70	47.4 (+/- 8.6)	NR (3)	BRCA1 (3)	NR (3)	NR (3)	Negative (3)	Peritoneal and omental biopsies (3): negative
Minig 2018 (25)	3/359	56.3 (+/- 5.7)	Breast (1)	BRCA1 or 2 (3)	Normal (3)	Normal (3)	Negative (2) Positive (1)	
Poon 2016 (26)	3/138	52.3 (49-57)	Breast (1)	BRCA1 (2) BRCA2 (1)	NR (3)	NR (3)	Negative (1) Positive (atypical cells) (1) ND (1)	
Powell 2013 (27)	16/407	52.5 (47.5-61.8)	NR (16)	BRCA1 (13) BRCA2 (3)	Normal (14) ND (2)	Normal (11) ND (2) Ovarian cyst (2) Hydrosalpinx (1)	Negative (13) Positive (3)	
Reitsma 2013 (28)	3/360	54.3 (+/- 3.8)	Breast (1)	BRCA2 (2) BRCA2 UV (1)	Normal (3)	Normal (3)	Negative (3)	
Ricciardi 2017 (29)	7/411	54 (43-67)	Breast (6)	BRCA1 (7)	Normal (7)	Normal (7)	Negative (7)	
Rudaitis 2020 (30)	7/71	45 (43-52)	Breast (1)	BRCA1 (7)	ND (7)	Normal (7)	NR/ND (7)	
Rush 2020 (31)	9/644	47 (42.5-57.5)	Breast (4)	BRCA1 (6) BRCA2 (2) PALB2 (1)	Normal (9)	NR (9)	Negative (7) Positive (2)	TLH (5) TAH (2)
Selmes 2015 (32)	1/93	40	Breast (1)	BRCA1 (1)	NR (1)	NR (1)	ND (1)	
Van der Hoeven 2018 (19)	2/235	56.5 (+/- 26.2)	Breast (1)	BRCA1 (2)	Normal (2)	Normal (2)	ND (2)	
Wethington 2013 (6)	12/593	48.5 (44.3-66.5)	Breast (2)	BRCA1 (5) BRCA2 (4) BRCA2 rearrangement (1) Unknown, but high risk (2)	Normal (12)	Normal (10) ND (2)	Negative (11) Positive (1)	Serous adenofibroma (1) Endosalpingiosis (1)
Zakhour 2016 (33)	9/257	57.1 (49.5-66.5)	No (9)	BRCA1 (8) BRCA2 (1)	Normal (12)	Normal (12)	Negative (7) Negative: Atypic cells (1) ND (1)	HE (2)
Low-risk cohort								
Chay 2016 (12)	5 (unknown)	52 (48.5-63)	Breast (1) NR (3)	BRCA1 (1) NR (4)	NR (4) Elevated (1)	Ovarian mass (1) Ovarian cyst (1)	NR (1) ND (4)	USO + USE (1): ovarian fibroma (1) TAH (2); Ovarian cyst (1) Endometriosis(2) Hydrosalpinx(1)
Morrison 2015 (34)	3 (unknown)	58 (+/- 7.2)	NR (3)	NR (3)	NR (3)	NR (3)	NR (3)	HE (3) Uterine leiomyomas (2)
Rabban 2014 (35)	3/522	64 (+/- 18.5)	No (3)	Negative (1) ND (2)	NR (3)	Adnexal cyst (3)	NR/ND (3)	USO (1)
Tomasch 2020 (36)	1/98	57	NR (1)	Unknown (1)	NR (1)	NR (1)	NR (1)	Cholecystectomy and bilateral prophylactic salpingectomy

HE, hysterectomy; ND, not done; NR, not reported; RRSO, risk reducing salpingo-oophorectomy; STIC, serous tubal intraepithelial carcinoma; TAH, total abdominal hysterectomy; TLH, total laparoscopic hysterectomy; USE, unilateral salpingectomy; USG, ultrasound scan test; USO, unilateral salpingo-oophorectomy; UV, unknown variant. If possible, median age with interquartile range was calculated.

**Table 2:** Follow-up of patients with STIC included in our systematic review in alphabetical order (white: high-risk cohort; grey: low-risk cohort).

Reference	STIC cases with follow-up	Surgical staging after positive washings at RRSO	Surgical staging after negative washings at RRSO	Surgical staging after unreported or nor performed washings at RRSO	Chemo-therapy	Median follow-up (range) or mean (+/- SD) in months	Status at follow-up	Recurrence	Additional information on recurrences	Additional information
**High-risk cohort**										
Blok 2019 (13)	4		ND (2)	ND (2)	ND (4)	62.1 (3.1-131.3)	Alive (2) NED (2)	PPSC (2)	Recurrence after 80 and 118 months: both with unknown cytology at RRSO, no staging or chemotherapy thereafter	
Carcangiu 2006 (20)	3		ND (2)	ND (1)	ND (3)	44 (+/- 40.3)	NED (3)			
Conner 2014 (21)	11		Surgical staging (3) (no details): negative ND (3) ND (5)		6 cycles C/P (2) 2 cycles C/P (1) ND (8)	60 (24-84)	Alive (11)	Yes (1)	Elevated serum CA125 and ascites (1) 48 months after RRSO, but no tissue diagnostic	
Gornjec 2020 (22)	3		Surgical staging (3): negative		ND (3)	29 (15-51)	NED (3)			Staging procedure not described
Lamb 2006 (23)	4	“second look operation” (1): negative	ND (3)		6 cycles C/P (1; positive washing) 3 cycles C/P (1)	28 (unkown)	NED (4)			
Miller 2017 (24)	3	–	NR (3)		NR (3)	32.5 (+/- 24.7)	NED (3)			Data for cohort, not exclusively for STIC patients
Minig 2018 (25)	3	OE, PPALND(1): negative	ND (2)		ND (3)	23 (+/- 10.8)	NED (3)			
Poon 2016 (26)	3	Offered (1): Surgical staging versus observation: Patient opted for observation with CA-125 and clinical review 6/12 for 3.5 years. Patient discharged to LMO with yearly CA-125.	ND (1), Observation	Offered (1): Surgical staging versus observation. Patient opted for observation with yearly CA-125.	ND (3)	79.3 (+/- 31.9)	NED (3)			
Powell 2013 (27)	16	Staging surgery without lymph node excision (2)/with lymph node excision (1)	Staging surgery without lymph node excision (4)/with lymph node excision (2)		ND (12) 6 cycles C/P (2, positive washing) 3 cycles C/P (2, negative washing, no surgical staging)	79 (59.5-100.5)	NED (15)	Yes (1)	43 months after RRSO: omental deposits	
Reitsma 2013 (28)	3		ND (3)		NR (3)	12 (+/- 12.5)	NED (3)			
Ricciardi 2017 (29)	7		Surgical staging (4): laparoscopic HE, OE, random peritoneal biopsies and peritoneal washing: No malignancies		ND (7)	30 (9-84)	NED (7)			
Rudaitis 2020 (30)	7	NR/ND	NR/ND	NR/ND	ND (7)	54 (37.2-63.6)	NED (7)			
Rush 2020 (31)	9	NR	NR		6 cycles C/P (2; positive washings) 3 cycles C/P (2; negative washings)	144 (42-192)	NED (9)			
Selmes 2015 (32)	1			NR (1)	NR(1)	22	NED (1)			
Van der Hoeven 2018 (19)	2			ND (2)	ND (2)	78 (59-96)	Dead of disease (1)	Yes (1)	36 months after RRSO, died 59 months after RRSO at disease Deceased due to breast cancer (1)	
Wethington 2013 (6)	12	Surgical staging (1): TAH, omentectomy, biopsies: negative	TH(7), OE (7) Peritoneal biopsies (6) Diaphragm biopsies (3) PLND (1) PPALND (5) Surgical staging declined (3) or performed outside the hospital (1): negative		ND (12)	28 (20-33.8)	NED (12)			4 patients had additional postoperative imaging: normal
Zakhour 2016 (33)	9		ND (8)	ND (1)	ND (9)	81.3 (38.5-109.5)	NED (9)	PPSC (2)	Recurrences after 32 and 42 months: both with negative cytology and negative HE at RRSO, no staging or chemotherapy thereafter	
**Low-risk cohort**										
Chay 2016 (12)	5			Surgical staging (1): vaginal HE, peritoneal washing, OE, PPALND sampling: no malignancies	ND (5)	25 (11.5-83)	NED (5)	No (5)		2° diagnosis of TNBC (BRCA1 mutation) 2° diagnosis of colon cancer (HGSC)
Morrison 2015 (34)	3			ND (3)	4 cycles C/P (1)	6.7 (+/- 4.6)	NED (3)	No (3)		Non-prophylactic setting
Rabban 2014 (35)	3			Surgical staging (1): HE, completion salpingo-oophorectomy, lymph node dissection, OE: No malignancies	ND (3)	14 (+/- 10.1)	NED (3)		–	
Tomasch 2020 (36)	1			ND (1)	ND (1)	28	Peritoneal carcinomatosis (HGSC)	Yes (1)		Prophylactic salpingectomy at the time of elective laparoscopic cholecystectomy. No clinical data available. STIC was overseen and later on detected with the SEE-FIM protocol.

C/P, Carboplatin/Paclitaxel; HE, hysterectomy; HGSC, high-grade serous cancer; LAVH, laparoscopic-assisted vaginal hysterectomy; LMO, local medical officer; NED, no evidence of disease; OE, omentectomy; PLND, pelvic lymph node dissection; PPALND, pelvic and paraaortic lymph node dissection; PPSC, primary peritoneal serous carcinoma; RRSO, risk-reducing salpingo-oophorectomy, SEE-FIM, Sectioning and Extensively Examining the FIMbria; STIC, serous tubal intraepithelial carcinoma; TNBC, triple negative breast cancer.

**Table 3:** Overview of the 112 included STIC patients.

Characteristics	112 patients
Median age (years)	52.3 (46.3-60)
BRCA status (%) -BRCA1 -BRCA2 -BRCA1 or 2 -Low risk/unknown	71 (63.4) 18 (16.1) 8 (7.1) 9 (8.0)
Peritoneal washing at RRSO/surgery (%) -Negative -Positive -Atypical cells -Not done/not reported	70 (62.5) 9 (8.0) 1 (0.9) 32 (28.6)
Surgical staging (%) -Performed -Not done/not reported	32 (28.6); no malignancies detected 80 (71.4)
Adjuvant treatment (%) -Chemotherapy	14 (12.5)
Recurrence (%)	8 (7.1)
Median time to recurrence with interquartile range (months)	42.5 (33-72)
Time to latest recurrence (months)	118
Risk for HGSC (%) -5 years -10 years	10.5 21.6
Median follow-up (months)	36 (25.3-84)

HGSC, high-grade serous carcinoma; RRSO, risk-reducing salpingo-oophorectomy; STIC, serous tubal intraepithelial carcinoma.

The surgical staging procedures mostly included omentectomy and in some cases a pelvic and paraaortic lymph node dissection (see **Table 1**).

In the study of Wethington and colleagues, all patients with an isolated STIC were offered a surgical staging, including hysterectomy, omentectomy and in five cases pelvic and paraaortic lymph node dissections. All procedures were without pathological findings. Three patients declined a surgical staging (6). Postoperative imaging as staging was hardly reported. Four out of 12 patients in the cohort of Wethington had an additional postoperative imaging without pathological findings (6).

14 out of 112 (12.5%) patients received adjuvant chemotherapy consisting of a combination of Carboplatin and Paclitaxel. Five out of nine patients with a positive washing received chemotherapy as well as seven patients with a negative cytology and one patient with a non-reported cytology. Follow-up mostly included clinical observation with CA-125 yearly. Pooled median follow up was 36 months (IQR: 25.3-84).

**Figure 2:**
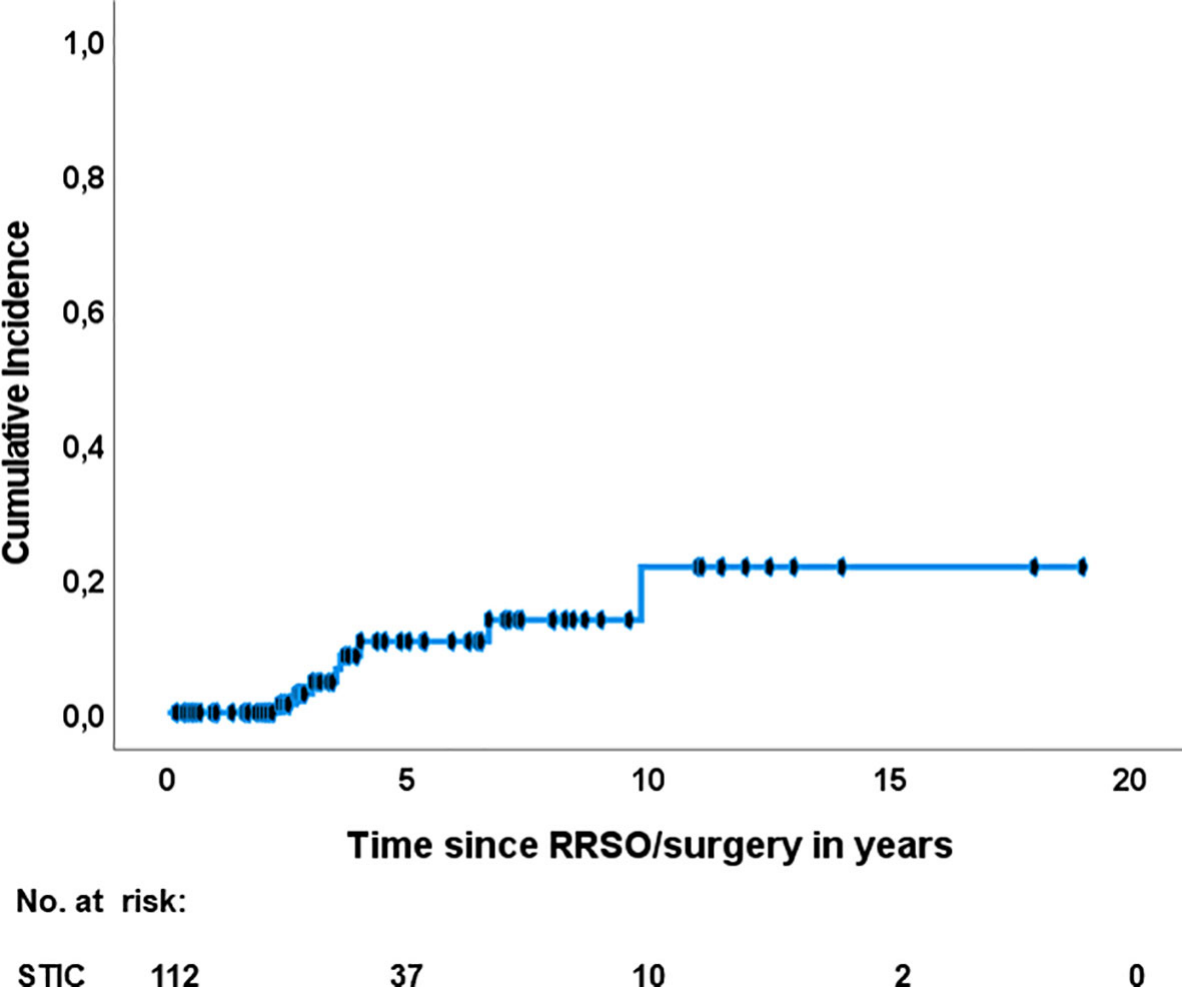
Recurrence of HGSC after isolated STIC diagnosis. RRSO risk reducing salpingo-oophorectomy; STIC, serous tubal intraepithelial carcinoma.

Eight out of 112 patients developed a subsequent HGSC (7.1%, 95% CI 2.3-12%), listed in **Table 4**. Pooled median time to recurrence were 42.5 (IQR: 33-72) months. The five (ten)- year- HGSC rate was 10.5% (21.6%), determined by the Kaplan-Meier estimation (**Figure 2**). The latest HGSC recurred 118 months after the diagnosis of STIC at RRSO/surgery. Seven out of eight patients were *BRCA1* carriers and one patient had an unknown *BRCA* status since STIC was detected after revaluation of a salpingectomy during cholecystectomy (36). No *BRCA2* carrier presented a recurrence in the selected studies. A recurrence occurred in four patients with a negative peritoneal washing, in three patients in which no pelvic washing was done and in one patient without a reported peritoneal cytology at the time of the first surgery.

**Table 4:** Characteristics of the eight STIC patients with subsequent HGSC.

Patient number	Age at RRSO/surgery	Menopausal status at RRSO/surgery	*BRCA* gene involved	Peritoneal washing	Additional procedures at RRSO/surgery	Surgical staging	Adjuvant treatment	Recurrence	Time to recurrence(months)
1 (Blok 2019) (13)	46	premenopausal	BRCA1	ND	ND/NR	ND	ND	PPSC	118
2 (Blok 2019) (13)	53	premenopausal	BRCA1	ND	ND/NR	ND	ND	PPSC	80
3 (Conner 2014) (21)	46	NR	BRCA1	negative	ND	ND	ND	Elevated serum CA125 and ascites	48
4 (Powell 2013) (27)	49	NR	BRCA1	negative	HE	ND	ND	Omental deposits	43
5 (Tomasch 2020) (36)	57	NR	Low risk (unknown)	ND	Cholecystectomy	ND	ND	Peritoneal carcinomatosis (HGSC)	28
6 (Van der Hoeven, 2018) (19)	54	NR	BRCA1	NR	ND	ND	ND	Recurrence at peritoneum, omentum, uterus	36
7 (Zakhour 2016) (33)	50	NR	BRCA1	negative	HE	ND	ND	PPSC	97
8 (Zakhour 2016) (33)	60	NR	BRCA1	negative	ND	ND	ND	PPSC	104

HE, Hysterectomy; HGSC, high-grade serous carcinoma; PPSC, primary peritoneal serous carcinoma; ND, not done; NR, not reported; RRSO, risk-reducing salpingo-oophorectomy; STIC, serous tubal intraepithelial carcinoma.

### Risk of bias assessment

The risk of bias assessment is shown in appendix B. In 10/21 (48%) studies, STIC was diagnosed according to predefined pathological criteria. 17/21 (81%) studies reported the mutation status for the cohort. 11/21 (52%) studies operated according to a predefined protocol and only one study had a predefined treatment protocol for STIC. In general, adjuvant treatment was adequately described in 13/21 (61%) studies. Two studies had a predefined protocol for the follow-up of patients with STIC. Finally, 18/21 (86%) studies reported an adequate follow-up for patients with STIC.

## Discussion

### Summary of main results and results in the context of published literature

In our review, the rate for subsequent HGSC after an isolated STIC diagnosis was 7.1%. In literature, recurrence rates in patients with isolated STIC ranged from 0-22% (21, 26, 28, 33), mostly due to small numbers of patients per study. One systematic review reported a rate of 4.5% in 2015 (7) and a more recent one in 2018 a rate of 11% (19). Our rate of recurrence may be more accurate since all patients with isolated STIC and available follow-up of the current literature were included. It is important to note that our rate might be probably increased with a longer follow-up of patients after an isolated STIC diagnosis, because the pooled median follow-up was 36 months and the pooled recurrence was detected more than half a year later after 42.5 months. A long follow-up is necessary to be able to determine the real incidence of HGSC. Our study determined a high and clinically relevant cancer risk for HGSC after STIC diagnosis of 10.5% (21.6%) after five (ten) years according to the Kaplan-Meier estimation. This again underlines the importance of a long follow-up, especially if we consider that the latest recurrence occurred almost 10 years after initial surgery. During the preparation of the manuscript, Steenbeek and colleagues published a systematic review about the risk of peritoneal carcinomatosis after RRSO with similar results in February 2022. They report a five- and ten- year- risk of developing peritoneal carcinomatosis of 10.5% and 27.5% after RRSO, respectively (37). Due to the prior closure of our data collection, we could not include their newly published STIC cases.

Interestingly, only *BRCA1* carriers developed a subsequent HGSC. In general, *BRCA1* carriers have the highest risk of occult neoplasia at RRSO (31). For all *BRCA* mutation carriers, a 3.5% cumulative risk for peritoneal cancer after prophylactic oophorectomy was reported after 20 years of follow-up (38). One STIC patient had a *PALB2* gene mutation which is also involved in hereditary breast and ovarian cancer but insufficiently determines the ovarian cancer risk (39, 40).

### Strengths and weaknesses

We present a comprehensive review on published clinical outcomes and treatment modalities of patients with isolated STIC. Our strength is that our study contains the largest patient collective with isolated STIC and follow-up in the high-risk and especially the low-risk population so far. An increase in the number of STIC patients in the low-risk population is expected because opportunistic salpingectomies are recommended during routine surgery to prevent epithelial ovarian cancer (10).

However, our study reanalysed published data. The quality of collected data was low and with significant risk of bias (see appendix B). The latter included incomplete clinical data, heterogeneous follow-up data, e.g. only the mean data was given, the lack of data regarding a standard diagnostic, staging, treatment and surveillance. An important confounder in many studies was the short follow-up period after the STIC diagnosis, which might disguise the real rate of subsequent HGSC after RRSO/salpingectomy.

The impact of STIC in a low risk population is difficult to assess due to the lack of information. A Canadian study reported STIC in eight out of 9392 women (<0.01%) with benign diagnoses who had a normal risk of ovarian cancer using the SEE-FIM protocol (9). However, the SEE-FIM protocol was not routinely applied in non-RRSO surgery in the past and therefore published data on the incidence of STIC low-risk populations should be interpreted with caution. In general, diagnosing STIC is challenging with only moderate reproducibility. A recently published systematic review suggests not only the use of the SEE-FIM protocol, but also evaluation by a subspecialized pathologist, rational use of immunohistochemical staining, and obtaining a second opinion from a colleague to secure the diagnosis (41). Furthermore, there can also be a HGSC unrelated to a STIC diagnosis. Another bias is that STIC patients with positive washings were included to maintain the comparability to previous studies (7). However, not every patient received a subsequent surgical staging to eliminate the risk of a HGSC. The impact of positive peritoneal washings remains unclear as well. The routine use of peritoneal biopsies during RRSO does not seem to improve the detection of occult malignancies (42). In our review, nine patients had a positive washing and six underwent surgical staging without pathological findings. No patient with a positive washing developed a recurrence. According to the study of Wethington 15% of the peritoneal washings were positive at the time of RRSO and therefore recommended as a component of RRSO (6).

### Implications for practice and future research; conclusion

Clinical management of STIC is still a matter of debate. It is important that patients are informed about their potential risk of developing pelvic HGSC after a STIC diagnosis. A surgical staging should be considered (43), especially in cases of a positive peritoneal washing at initial RRSO/surgery to reduce the risk of synchronous HGSC. A surgical staging mostly included hysterectomy, omentectomy, pelvic and paraaortic lymph node dissection and peritoneal washing in the published studies. In case of a positive peritoneal washing at initial surgery, which implies circulating malignant cells in the peritoneal cavity, some institutions offered adjuvant chemotherapy. The latter usually comprised six cycles of Carboplatin and Paclitaxel. However, if the surgical staging is without evidence of disease, observation remains a reasonable option and avoids possible chemotherapy-induced adverse events (6). Adjuvant chemotherapy for intraepithelial neoplasia is not recommended any longer (31, 43). A radiological staging was rarely reported in our study.

Routine surveillance is recommended for the next years of follow-up, because the time from STIC to invasive cancer has been suggested to be approximately seven years and has guided the recommendation for RRSO in *BRCA1* patients at the age of 35–40 years (44). This is coherent with the findings of Stanciu and colleagues who published seven cases with isolated STICs. Two of these patients (28%) developed peritoneal HGSC within 53 and 75 months after RRSO. The publication was not included in our study, because the follow-up of the five other patients with isolated STIC was missing (45).

To date, no effective screening tool exists to monitor STIC patients (26). Most of the published studies included annual clinical check-ups with pelvic ultrasound and in some cases routine evaluation of serum CA-125. *BRCA* status should be checked in cases of isolated STIC as well. No routine screening for ovarian HGSC should be offered to women of the general population. Two prospective randomized trials could not reduce ovarian cancer mortality with simultaneous screening of CA-125 and transvaginal ultrasound compared with usual care in the normal population (46, 47).

To summarize, several questions concerning STIC remain unclear and the therapy may require an individualized treatment plan. We are urgently in need of registries for longer follow-up data of STIC patients to assess the real incidence of HGSC after a STIC diagnosis. Future multicentre and international efforts are needed to generate a large cohort of patients with STIC to allow further subgroup analyses, e.g. regarding histopathological characteristics. In the meantime, systematic reviews will help to gather information and to define and update guidelines for the management of STIC.

## Data availability statement

The original contributions presented in the study are included in the article/**Supplementary Material**. Further inquiries can be directed to the corresponding author.

## Author contributions

Conceptualization: VL, AL, AH and MB. Design: VL. and AL. Data acquisition: VL, AL and MB. Analysis and interpretation: VL, AL and MB. Writing- original draft of the manuscript: VL. Writing - review and editing: VL, AL, JV, AH and MB. All authors have read and agreed to the published version of the manuscript. All authors contributed to the article and approved the submitted version.

## Acknowledgments

We would like to thank Lorena Cascant Ortolano from the library of the Johannes Gutenberg- University Mainz for her support in conducting the literature search.

## Conflict of interest

The authors declare that the research was conducted in the absence of any commercial or financial relationships that could be construed as a potential conflict of interest.

## Publisher’s note

All claims expressed in this article are solely those of the authors and do not necessarily represent those of their affiliated organizations, or those of the publisher, the editors and the reviewers. Any product that may be evaluated in this article, or claim that may be made by its manufacturer, is not guaranteed or endorsed by the publisher.
