# Supplementary material for: Incidence of pelvic high-grade serous carcinoma after isolated STIC diagnosis: A systematic review of the literature
Source: Front Oncol. 2022 Aug 31;12:951292. doi: 10.3389/fonc.2022.951292 (PMC9472545; doi:10.3389/fonc.2022.951292)
Supplement: Supplementary file 1 [file DataSheet_1.pdf]

Supplementary Material

Appendix A: Search strategy

Database	ID	Search
Medline Ovid	1	(((exp Cystadenocarcinoma, Serous/ or Carcinoma in Situ/ or ((intraepithelial or intra-epithelial or preinvasive) adj3 (carcinoma* or neoplasm* or cancer*)).ab,ti.) and (Fallopian Tube Neoplasms/ or *Ovarian Neoplasms/ or Carcinoma, Ovarian Epithelial/ or ((Fallopian or high grade ovarian) adj3 (carcinoma* or neoplasm* or cancer*)).ab,ti.)) or (STICs or STIN or ((serous or tubal) adj3 (intraepithelial or intra-epithelial) adj3 (carcinoma* or neoplasm* or cancer*))).ab,ti.) not (animals not humans).sh.
	2	limit 1 to yr="2006 -Current"
Web of Science	7	(#1) AND #5
	6	(#1) AND #5
	5	#4 OR #3 OR #2
	4	TS=((serous or tubal) near/3 (intraepithelial or intra-epithelial) near/3 (carcinoma* or neoplasm* or cancer*))
	3	TS=(STICs or STIN)
	2	TS=((Fallopian or “high grade ovarian”) near/3 (carcinoma* or neoplasm* or cancer*))
	1	TS=((intraepithelial or intra-epithelial or preinvasive) near/3 (carcinoma* or neoplasm* or cancer*))
Cochrane	#1	MeSH descriptor: [Cystadenocarcinoma, Serous] explode all trees

	#2	MeSH descriptor: [Carcinoma in Situ] this term only
	#3	((intraepithelial OR intra-epithelial or preinvasive) NEAR/3 (carcinoma* or neoplasm* or cancer*)):ti,ab,kw
	#4	{OR #1-#3}
	#5	MeSH descriptor: [Fallopian Tube Neoplasms] explode all trees
	#6	MeSH descriptor: [Ovarian Neoplasms] this term only
	#7	MeSH descriptor: [Carcinoma, Ovarian Epithelial] explode all trees
	#8	((Fallopian or “high grade ovarian”) NEAR/3 (carcinoma* or neoplasm* or cancer*)):ti,ab,kw
	#9	(STICs or STIN):ti,ab,kw
	#10	((serous or tubal) NEAR/3 (intraepithelial or intra-epithelial) NEAR/3 (carcinoma* or neoplasm* or cancer*)):ti,ab,kw
	#11	{OR #5-#10}
	#12	#4 AND #11 with Publication Year from 2006 to 2021, with Cochrane Library publication date Between Jan 2006 and Jul 2021, in Trials

Appendix B: Risk of bias assessment for each study (alphabetical order) included in the systematic review on the basis of Van der Hoeven et al. 2018 (20)

(1): since 2010: SEE-FIM protocol; (2): low-risk population; (3): only PPSC absence or presence described at follow-up

HGSC: high-grade serous carcinoma; PPSC: primary peritoneal serous carcinoma; ND: not done; NR: not reported; SEE-FIM: Sectioning and Extensively Examining the FIMbria; STIC: serous tubal intraepithelial carcinoma

**Reference**	**Diagnosis of STIC**		**Reporting bias**			**Indication bias**		**Follow- up**			
	**According to predefined cirteria**	**By an expert pathologist**	**Original cohort size described**	**Mutation status of cohort described**	**Median/mean age of the cohort described**	**RRSO/surgery according to a predefined protocol**	**Treatment of STIC according to a predefined protocol**	**Adjuvant treatment of STICs described**	**Follow-up according to a predefined protocol for cohort**	**Adequate follow-up reported for cohort**	**Adequate follow-up reported for STIC cases**
Blok 2019 (38)	No (1)	Yes	Yes	Yes	Yes	No	No	Yes	No	Yes	Yes
Caracangiu 2006 (39)	No	No	Yes	Yes	Yes	No	No	No	Yes	Yes	Yes
Chay 2016 (22)	No	No	Yes	No (2)	Yes	No	No	No	No	No	No
Conner 2014 (40)	Yes	No	Yes	Yes	Yes	No	No	No	No	No	Yes

Gornjec 2020 (41)	Yes	Unkown	Yes	Yes	Yes	Yes	Yes	Yes	Yes	No	Yes
Lamb 2006 (42)	No	Yes	Yes	Yes	Yes	Yes	No	Yes	No	Yes	Yes
Miller 2017 (43)	No	Yes	Yes	Yes	Yes	Yes	No	No	No	Yes	No
Minig 2018 (44)	Yes	Yes	Yes	Yes	Yes	Yes	No	Yes	No	Yes	Yes
Morrison 2015 (23)	No	Yes	Yes	No (2)	Yes	No	No	Yes	No	Yes	Yes
Poon 2016 (26)	No	Yes	Yes	Yes	Yes	No	No	Yes	No	No	Yes
Powell 2013 (45)	No	No	Yes	Yes	Yes	No	No	Yes	No	No	Yes
Rabban 2014 (24)	No	Yes	Yes	No (2)	Yes	No	No	Yes	No	No	Yes
Reitsma 2013 (29)	Yes	Yes	Yes	Yes	Yes	No	No	No	No	Yes	Yes
Ricciardi 2017 (46)	Yes	Unkown	Yes	Yes	Yes	Yes	No	Yes	No	No	Yes
Rudatitis 2020 (47)	Yes	Yes	Yes	Yes	Yes	Yes	No	No	No	No	Yes
Rush 2020 (21)	Yes	Unkown	Yes	Yes	Yes	Yes	No	Yes	No	No	Yes
Selmes 2015 (48)	Yes	Yes	Yes	Yes	Yes	No	No	Yes	No	Yes	Yes

Tomasch 2020 (25)	No	No	Yes	No (2)	Yes	Yes	No	No	No	No	No
Van der Hoeven 2018 (20)	Yes	Yes	Yes	Yes	Yes	Yes	No	Yes	No	Yes	Yes
Wethington 2013 (27)	Yes	Yes	Yes	Yes	Yes	Yes	No	Yes	No	No	Yes
Zakhour 2016 (28)	No	Yes	Yes	Yes	Yes	Yes	No	No	No	Yes	Yes (3)
Total	10/21<br>(48%)	13/21<br>(62%)	21	17/21<br>(81%)	21	11/21<br>(52%)	1/21<br>(5%)	13/21<br>(62%)	2/21<br>(10%)	10/21<br>(48%)	18/21<br>(86%)
